# Assessment of potential effects of the electromagnetic fields of mobile phones on hearing

**DOI:** 10.1186/1471-2458-5-39

**Published:** 2005-04-19

**Authors:** Ingrida Uloziene, Virgilijus Uloza, Egle Gradauskiene, Viktoras Saferis

**Affiliations:** 1 Institute for Biomedical Research of Kaunas University of Medicine, Eiveniu 4, Kaunas, Lithuania; 2 Department of Otolaryngology, Kaunas University of Medicine, Eiveniu 2, Kaunas, Lithuania

## Abstract

**Background:**

Mobile phones have become indispensable as communication tools; however, to date there is only a limited knowledge about interaction between electromagnetic fields (EMF) emitted by mobile phones and auditory function. The aim of the study was to assess potential changes in hearing function as a consequence of exposure to low-intensity EMF's produced by mobile phones at frequencies of 900 and 1800 MHz.

**Methods:**

The within-subject study was performed on thirty volunteers (age 18–30 years) with normal hearing to assess possible acute effect of EMF. Participants attended two sessions: genuine and sham exposure of EMF. Hearing threshold levels (HTL) on pure tone audiometry (PTA) and transient evoked otoacoustic emissions (TEOAE's) were recorded before and immediately after 10 min of genuine and/or sham exposure of mobile phone EMF. The administration of genuine or sham exposure was double blind and counterbalanced in order.

**Results:**

Statistical analysis revealed no significant differences in the mean HTLs of PTA and mean shifts of TEOAE's before and after genuine and/or sham mobile phone EMF 10 min exposure. The data collected showed that average TEOAE levels (averaged across a frequency range) changed less than 2.5 dB between pre- and post-, genuine and sham exposure. The greatest individual change was 10 dB, with a decrease in level from pre- to post- real exposure.

**Conclusion:**

It could be concluded that a 10-min close exposure of EMFs emitted from a mobile phone had no immediate after-effect on measurements of HTL of PTA and TEOAEs in young human subjects and no measurable hearing deterioration was detected in our study.

## Background

Due to wide spread use of the Global System for Mobile Communications (GSM) mobile phones they have become indispensable as communication tools and therefore any consequent biological effects should be considered as a high-priority environmental health issue. However, to date, there is an inadequate knowledge on what biological systems could be affected by the use of these devices. Biological effects of radio-frequency electromagnetic fields (EMF) transmitted by mobile phones are still a matter of public and scientific discussion. Sensations of burning or warmth around the ear [1], headache [2], disturbance of sleep [3], alteration of cognitive functions and neural activity [4,5], as well as alteration of the blood-brain barrier and a relative decrease in regional cerebral blood flow have been reported as effects resulting from mobile phone use [6,7]. The potential tumorous effect of EMFs is still a subject of debates and research [8-11].

The hearing system is in the closest proximity to the device so that hearing is potentially the most affected target of thermal and non-thermal effects. Moreover, the hearing system and particularly the cochlear outer hair cells (OHC) are known to be highly sensitive to a great variety of exogenous and endogenous agents and externally applied electric and magnetic fields are known to be able to produce some hearing sensation [12]. Despite all these considerations and evidence, only recently, some studies have analyzed the effects of mobile phones on the auditory system [13,14]. However, the results are not completely consistent.

Only limited research data concerning interaction between EMF emitted by mobile phones and auditory function and possible impact on hearing, are available in the literature. The animal experiments using distortion product otoacoustic emissions (DPOAEs) did not show statistically significant changes on the OHC functionality of adult and developing rats exposed as long as 30 days 1–2 h per day to EMF at 900 MHz and 1800 MHz frequencies [15,16].

No measurable change in evoked otoacoustic emissions (OAEs) was detected and none of the subjects reported a deterioration in hearing threshold level after 10-min exposure to the EMFs emitted by mobile phones in a recent human study on possible effects of the EMF of mobile telephones on hearing [17]. Other studies based on the auditory brainstem response and middle latency response methods concluded that 30 min mobile phone use has no short-term adverse effects on the human auditory system [18,19]. The small amount of publications shows that there is a big gap in the knowledge of potential biological effects of cellular phone use on hearing.

The aim of the present study was to assess the acute potential changes in human hearing function as a consequence of exposure to low-intensity EMF's produced by mobile phones at frequencies of 900 and 1800 MHz and a sham-exposure under double-blind conditions as determined by changes in transient evoked otoacoustic emissions (TEOAEs) and hearing threshold levels (HTL) in pure tone audiometry (PTA).

## Methods

The protocol of the study was elaborated in the frame of European Commission 5^th ^Framework project "GUARD: potential adverse effects of GSM cellular phones on hearing".

The study group consisted of 30 healthy volunteers (mean age 23.6 ± 1.2 years; range 18 – 29 years) without any evidence of hearing or ear disorder. There were 18 males (mean age 22.8 ± 1.7 years; range 18 – 28 years) and 12 females (mean age 24.9 ± 1.8 years; range 18 – 29 years).

The participants satisfied the following inclusion criteria: age between 18 and 30 years, no history of otological disorder and/or familial hearing disorder, no self reported hearing difficulty or persistent tinnitus, no exposure to severe noise, no ototoxic drugs, no excess consumption of alcohol or drugs 24 hours prior the testing. Instrumental examination: normal appearance of tympanic membrane on otoscopy, hearing threshold levels (HTL) in both ears no worse that 20 dB(A) at any of the standard audiometric frequencies between 0.5 and 8 kHz (Interacoustics AC-40 audiometer, Denmark), no evidence of conductive hearing loss based on air-conduction and bone-conduction audiograms, normal tympanograms and acoustic reflexes present in both ears for stimulation using a 1 kHz tone at 100 dB HL (Interacoustics AT 235 h tympanometer, Denmark), presence of clear recordable TEOAE, defined as SNR greater than 6 dB in two or more half octave bands centred at 1, 2 and 3 kHz (Otodynamics ILO-88 system, London).

All participants attended two study sessions: genuine and sham exposures. The administration of genuine or sham exposure was double blind and counterbalanced in order. Genuine or sham EMF exposures were performed on separate days (at least 24 hours apart) with the tested participant and tester both blind to the condition being used.

The study session consisted of baseline audiological and TEOAE measurements, genuine or sham GSM mobile phone exposure, and followed immediate repeated audiological and TEOAE measurements. Post-exposure measurements were performed in the same order as pre-exposure for each participant.

Pure tone audiometry (PTA) consisted of air conduction using 2-dB steps in the test ear only. TEOAE measurement was used to record TEOAEs according to "linear" protocol using clicks at 80 dB SPL. (Analysis time was 20 ms, 260 stimuli). Each TEOAE measurement run included a minimum of 260 "sweeps". A TEOAE was defined as the response if its amplitude was more than 3 dB above the level of the noise floor. Reproducibility more than 60% was considered acceptable for the analysis at 3 successive frequency bands ranging from 1 to 3 kHz.

GSM exposure utilized the normal output of a consumer mobile phone (NOKIA 6310) at full power for 10 minutes. Fifteen participants received GSM exposure at 900 MHz (full power 2 W) and the other fifteen participants received GSM exposure at 1800 MHz (full power 1 W). The exposure consisted of speech at a typical conversational level via an insert earphone to one ear, plus GSM exposure in either genuine (test) or sham (control) conditions. All test were performed in a sound-treated 2.5 × 2.0 m booth.

The NOKIA 6310 GSM mobile phone without SIM card (checked for correct power output) was used for GSM exposure. Control for carrier frequency, output level, transmit/receive mode of the mobile phone was utilized using specialized PHOENIX software. Therefore the mobile phone was connected via serial data cable from the PC to the phone.

To simulate the normal use of a mobile phone the participants were simultaneously exposed to both GSM radiation and an acoustic stimulus (speech material 10 min of duration). However, to prevent any possible effects from using the speaker in the handset, the speech material was delivered via an insert phone (EAR tone 3A ABR). The insert phone was used without the ear tip inserted and, therefore, the tube was taped along the subject's jaw with the entrance of the tube placed at the tragal notch of the ear. The standard speech material duration of 10 minutes, read out by male speaker, was digitally recorded on minidisk (Sony MiniDisc Recorder MDS 101). Then the speech sample was filtered (Syntrilium "Cool Edit") amplifying the low frequencies of the speech to compensate to some extent for the frequency weighting of the tragal presentation, so that the speech did not sound too unnatural. Having re-recorded the filtered material, the speech sample was calibrated in 2-cc couples (Bruel & Kjer type 4152, Denmark) to the required level in order to produce a sound pressure level at the ear drum equivalent to free field speech level of approximately 60 dB(A). A sound replay system used to replay speech to the ear of the participant consisted of minidisk player, audiometer and insert earphone.

During the test session NOKIA 6310 mobile phone was fixed to the tested ear using special headband and positioning system, so that the centre of the radiated field should be over the entrance of the external ear canal. All parts of the positioning system were made by non-metallic plastic materials in order to avoid any perturbation of the EMF emitted by the mobile phone. The subjects tested were asked to perform an attention task so that they attend to the speech stimulus, such as counting the number of a specific word in the speech material. After the completion of the GSM exposure the participants were asked appropriate questions about the speech material and their experience of any possible subjective effect from the exposure.

The study has been acknowledged by An Independent Ethics Committee of Kaunas University of Medicine and is in compliance with the Helsinki Declaration.

### Statistics

A statistical analysis was performed with the SPSS 12.0 (Statistical Package for Social Sciences) for Windows. Means of the groups and standard errors of the means (SEM) for each parameter were obtained for the genuine and sham GSM exposure. Confidence interval (CI) of 0.95 was chosen for statistical evaluation and significance level of 0.05 was chosen for testing statistical hypotheses. As all the participants underwent four-times testing (pre/post genuine exposure and pre/post sham exposure), four dependent samples of repeated measures were obtained. Therefore, for the comparison of the means of the audiological parameters in the total group (n = 30) Repeated Measures Analysis of Variance was used. An Exact Friedman Test was used for the comparison of the means of the audiological parameters in the groups tested for 900 MHz and 1800 MHz EMF frequncies (n = 15 each), respectively.

## Results

The subjects tested in the study tolerated the EMF exposure of mobile phones quite well. There were no subjective complaints after the exposure.

**Figure 1:**
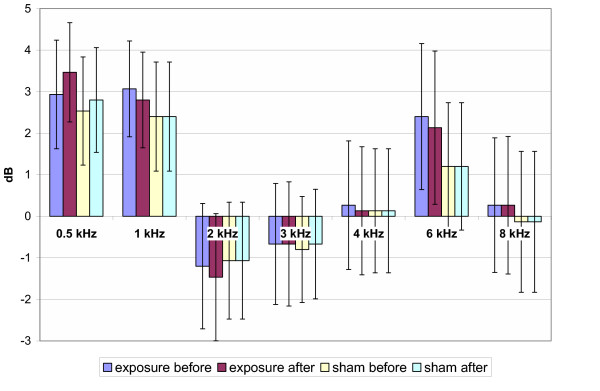
Hearing threshold levels (900 MHz exposure subgroup). Mean ± SEM, p > 0.05

**Figure 2:**
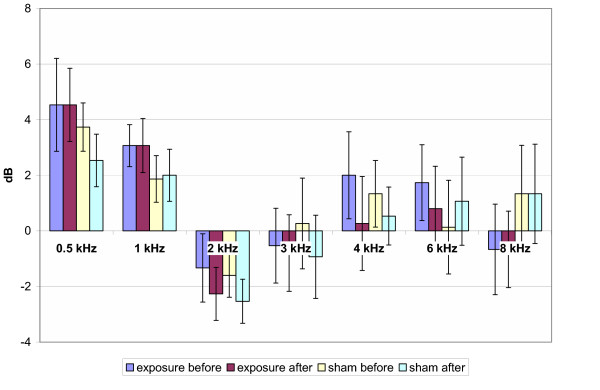
Hearing threshold levels (1800 MHz exposure subgroup). Mean ± SEM, p > 0.05

**Figure 3:**
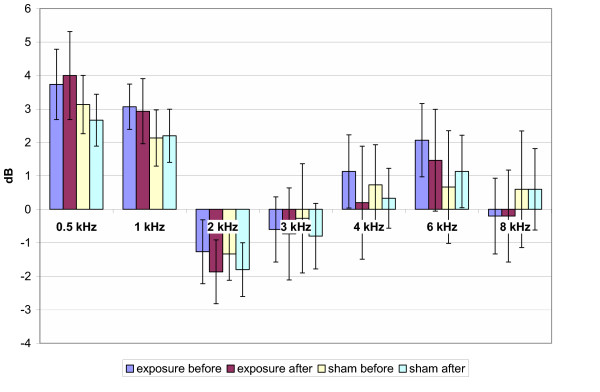
Hearing threshold levels (total group, n = 30). Mean ± SEM, p > 0.05

The means of air conduction HTL of PTA throughout the testing frequencies of pre/post genuine and pre/post sham GSM exposure are presented in Fig. 1 for 900 MHz exposure, in Fig. 2 for 1800 MHz exposure and in Fig. 3 for the total group, respectively. The analysis of means of HTL of the PTA with the Repeated Measures Analysis of Variance and Exact Friedman Test did not reveal any statistically significant differences between pre/post genuine and pre/post sham exposure groups (p > 0.05).

**Figure 4:**
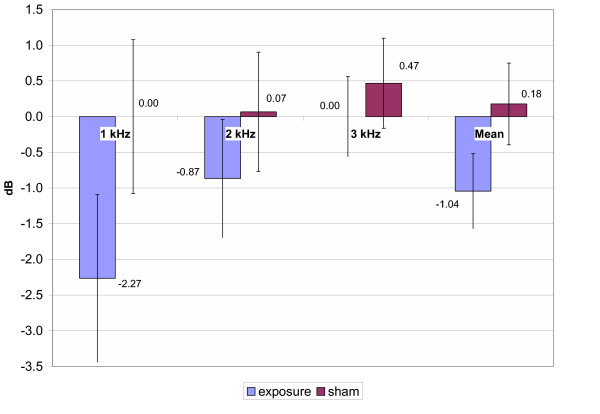
TEOAE amplitude shifts (900 MHz exposure subgroup). Mean ± SEM, p > 0.05

**Figure 5:**
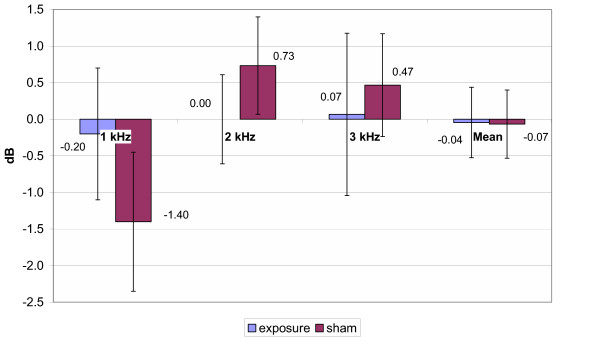
Mean TEOAE amplitude shifts (1800 MHz exposure subgroup, n = 15). Mean ± SEM, p > 0.05

**Figure 6:**
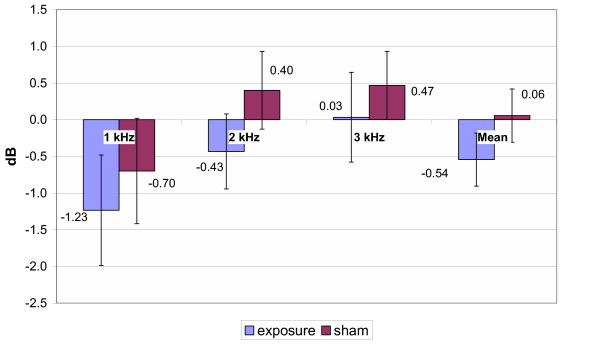
Mean TEOAE amplitude shifts (total group, n = 30,) Mean ± SEM, p > 0.05

As all of the subjects tested in the study had a normal hearing, recordings of TEOAEs were obtained (6–10 dB) above the noise floor through 1 – 3 kHz test frequency range for all sessions. Reproducibility of TEOAEs was >60% (91 ± 3 % in average). The mean amplitude shifts of the TEOAE measurements of pre/post genuine and pre/post sham GSM exposure are presented in Fig. 4 for 900 MHz exposure, in Fig. 5 for 1800 MHz exposure and in Fig. 6 for the total group, respectively. Statistical analysis with the Repeated Measures Analysis of Variance and Exact Friedman Test did not reveal any statistically significant differences in mean TEOAEs amplitude shifts between genuine and sham exposure groups (p > 0.05). The data collected showed that average TEOAE levels (averaged across a frequency range) changed less than 2.5 dB between pre- and post-, genuine and sham exposure. The greatest individual change was 10 dB, with a decrease in level from pre- to post- real exposure.

The results of the present study suggest that 10-min exposure to EMFs emitted by GSM mobile phone did not cause any detectable alterations in neither PTA nor TEOAEs.

## Discussion

GSM mobile phones have become very commonly used throughout the world within a short period of time. This has given rise to concern about potential influences of EMF emitted by mobile phones on health. Although there is no clear evidence to demonstrate harmful physiological effects of EMF at the levels used by mobile phones, however, there is a widespread public concern that there may be some potential harm [8-11,20-22].

Potential effects of mobile phone EMF radiation on hearing should be considered as one of the major priorities in the research of potential adverse effects of mobile phone use. Mobile phones are usually held in the closest proximity to the external ear and therefore, EMF exposure at the ear is high due to radiation from a remote earpiece. Since the cochlea is enclosed by very dense compact bone, located relatively deep, congested with the perilymph and endolymph, they all help to shield it from the mobile phone EMF [17]. However, the OHC of the inner ear are known to be the most sensitive and vulnerable elements of the auditory pathway. If subtle cochlear involvements occur, they might be detected through changes in TEOAEs, which directly reflect the function of cochlear OHC. Even minor changes in the functioning of OHCs, caused by various noxious factors, are known to considerably affect TEOAE amplitude [23-26]. On the other hand, TEOAEs represent acoustic responses of OHCs, which act like mechanoreceptor cells that generate force in their cell bodies to amplify sound and provide the exquisite sensitivity of the cochlea [27,29]. Consequently, the piezoelectric properties of OHCs that are essential for hearing might be relatively easily damaged by external EMFs emitted by mobile phones. Therefore, the present study in addition to conventional measures of HTL, which require a subjective response, employed an objective methodology of registration of TEOAE that is able to detect very small effects in hearing function with appropriate statistical power.

The experimental paradigm used in this study was the within-subject paradigm. This paradigm provided audiological measurements immediately before and immediately after exposure to EMF via a commercial mobile phone. As the procedure was conducted twice: one with a genuine exposure and one with a sham exposure, this approach maximized sensitivity to change, because between subjects' variations in the results were minimised by calculation of the difference between before and after measurements. A double-blind design of the procedure maximized objectivity of the experiment. EMF exposure dose used in the study was necessarily low but comparable with the use of the mobile phones in normal day life.

As stated above, statistical analysis of the results of the present study did not reveal any significant alterations of HTL after 10 min GSM mobile phone exposure. However, the main focus of this study was to analyse the effects of EMF of mobile phones on the TEOAE recorded before and immediately after a sham and a genuine exposure in a group of young subjects. The data collected showed that average TEOAE levels (averaged across a frequency range) changed less than 2.5 dB between pre- and post-, genuine and sham exposure. Therefore the variability of the TEOAE recorded before and after the GSM exposure and the individual variability appeared to be small and not statistically significant. This allows us to state that 10 min of EMF exposure at the maximum power (peak power: 1 W or 2 W according to the frequency) does not induce any measurable changes in the TEOAEs.

Some other unanswered questions of the present study should be mentioned. The measurements used in the study have been restricted by the frequency spectrum of the commercially available TEOAE equipment. Possibly, higher frequency instruments could be able to reveal more comprehensive information about the effects of EMF exposure [17]. As the study protocol was based on the comparison of the measures of the audiological tests obtained before and immediately after EMF exposure, only a relatively long-term or chronic alteration in hearing function could be detected by the present investigations. Some potential transitory, i.e. reversible, alterations in hearing function lasting for only a short time during the EMF exposure cannot be detected by these methods. Therefore, the simultaneous measurement of hearing function during the mobile phone's EMF emission would be of scientific interest. However, these implications could be considered as guidelines for further investigations.

## Conclusion

It could be concluded that a 10-min close exposure of EMFs emitted from a mobile phone had no immediate after-effect on measurements of HTL of PTA and TEOAEs in young adult human subjects and no measurable hearing deterioration at least at outer and middle ear and cochlear levels was detected in our study.

## Competing interests

The author(s) declare that they have no competing interests.

## Authors' contributions

IU has been the principal investigator, participated in study design and coordination and helped to draft the manuscript. VU participated in the planning of the study and coordinated the writing of the manuscript. VS performed the statistical analysis. EG participated in acquisition of audiological data. All authors contributed to the interpretation of results, have read and approved the final manuscript.

## Pre-publication history

The pre-publication history for this paper can be accessed here:


